# Improvement of left ventricular function assessment by global longitudinal strain after successful percutaneous coronary intervention for chronic total occlusion

**DOI:** 10.1371/journal.pone.0217092

**Published:** 2019-06-12

**Authors:** Misato Chimura, Shinichiro Yamada, Yoshinori Yasaka, Hiroya Kawai

**Affiliations:** Himeji Cardiovascular Center, Himeji, Japan; Osaka University Graduate School of Medicine, JAPAN

## Abstract

The benefit of revascularization of chronic total occlusion (CTO) in percutaneous coronary intervention (PCI) is controversial. On the other hand, left ventricular (LV) global longitudinal strain (GLS) is a more sensitive marker of LV myocardial ischemia and LV function than LV ejection fraction (EF). The purpose of this study was to investigate the impact of revascularization of CTO on LV function using LV GLS. A total of 70 consecutive patients (65.1±8.9 years, 59 males, LVEF 51.0±12.0%) with CTO who had a positive functional ischemia and underwent PCI, were included in this study. Echocardiography was performed before and 9 months after the procedure with conventional assessment including LV end-diastolic and end-systolic volume (LVEDV, LVESV), LVEF, and with 2DSTE analysis of GLS. Successful PCI was obtained in 60 patients (86%). There were no stent thromboses during follow-up. GLS showed a significant improvement 9 months after successful PCI (pre-PCI -12.4±4.1% vs. post-PCI -14.5±4.1%, P< 0.01), whereas in failed PCI group that did not change significantly (pre-PCI -13.2±4.2% vs. post-PCI -14.0±4.7%, P = 0.64). LVEF, LVEDV and LVESV did not change significantly during follow-up in both successful and failed groups. Successful PCI for CTO improved LV function, assessed by LV GLS.

## Introduction

Chronic total occlusions (CTO) are defined as lesions with TIMI (Thrombolysis in Myocardial Infarction) grade 0 flow for more than three months. CTO lesions are identified in 18.4% in patients undergoing elective percutaneous coronary intervention (PCI) in the absence of previous coronary artery bypass surgery or those presenting with acute myocardial infarction [1]. Several previous studies reported the effect of successful PCI for CTO, such as improvement of quality of life, exercise capacity, and reducing the need for late CABG surgery [2, 3]. However the benefits of revascularization using PCI for CTO are still controversial. Two-dimensional speckle-tracking echocardiography (2DSTE) is emerging as a novel technique to allow the assessment of LV systolic and diastolic function through the quantification of active myocardial deformation [4–6]. The global longitudinal strain (GLS) assessed with 2DSTE, which evaluates the longitudinal myocardial deformation, is more reproducible than left ventricular ejection fraction (LVEF) or wall motion score index (WMSI), is advantageous over the color kinesis technique and is proven to be effective in detecting the LV myocardial ischemia [7–13]. Moreover in patients with cardiovascular disease, myocardial dysfunction occurs even if overall LVEF is preserved, and that may be associated with impaired LV longitudinal deformation [14]. Accordingly, the purpose of this study was to investigate the impact of revascularization of CTO on LV function using LV GLS.

## Methods

### Patients

We conducted a retrospective study in a cohort of 70 consecutive patients with CTO who had attempted PCI at Himeji Cardiovascular Center between May 2009 and February 2014. All patients had single vessel coronary artery disease with CTO and had a positive functional ischemia study. We excluded patients who had an anticipated noncompliance with dual antiplatelet treatment for at least 12 months, the history of coronary artery bypass surgery, severe valvular disease, other co-morbid systemic disease and atrial fibrillation. CTO was defined as a coronary artery obstruction with thrombolysis in myocardial infarction (TIMI) grade 0 and all patients had a native vessel occlusion estimated to be of at least 3 months duration based on the time from diagnosis made on coronary angiography [15]. PCI and stent implantation were performed in a standard manner. Drug-eluting stents (DESs) were used in all of the PCI procedures. After the PCI, all of the patients were prescribed lifelong aspirin and clopidogrel for at least 12 months. Collateral channels and their Rentrop classification were analyzed in the pre-procedural coronary angiography. Successful PCI was defined as follows; the residual stenosis < 50% by visual estimation, a restoration of TIMI flow 3 in the target vessel after stent implantation and no immediate angiographic complications. Failed PCI was defined as failure to cross the occlusion or reduce obstruction to less than 50% in the target CTO [16].

All of the patients underwent an extensive baseline clinical history taking and physical examination, 12-lead electrocardiography, and transthoracic echocardiography. This study was approved by the research ethics committee of Himeji Cardiovascular Center and carried out in accordance with approved guidelines. Written informed consent was obtained from all patients.

### Transthoracic echocardiography

**Fig 1:**
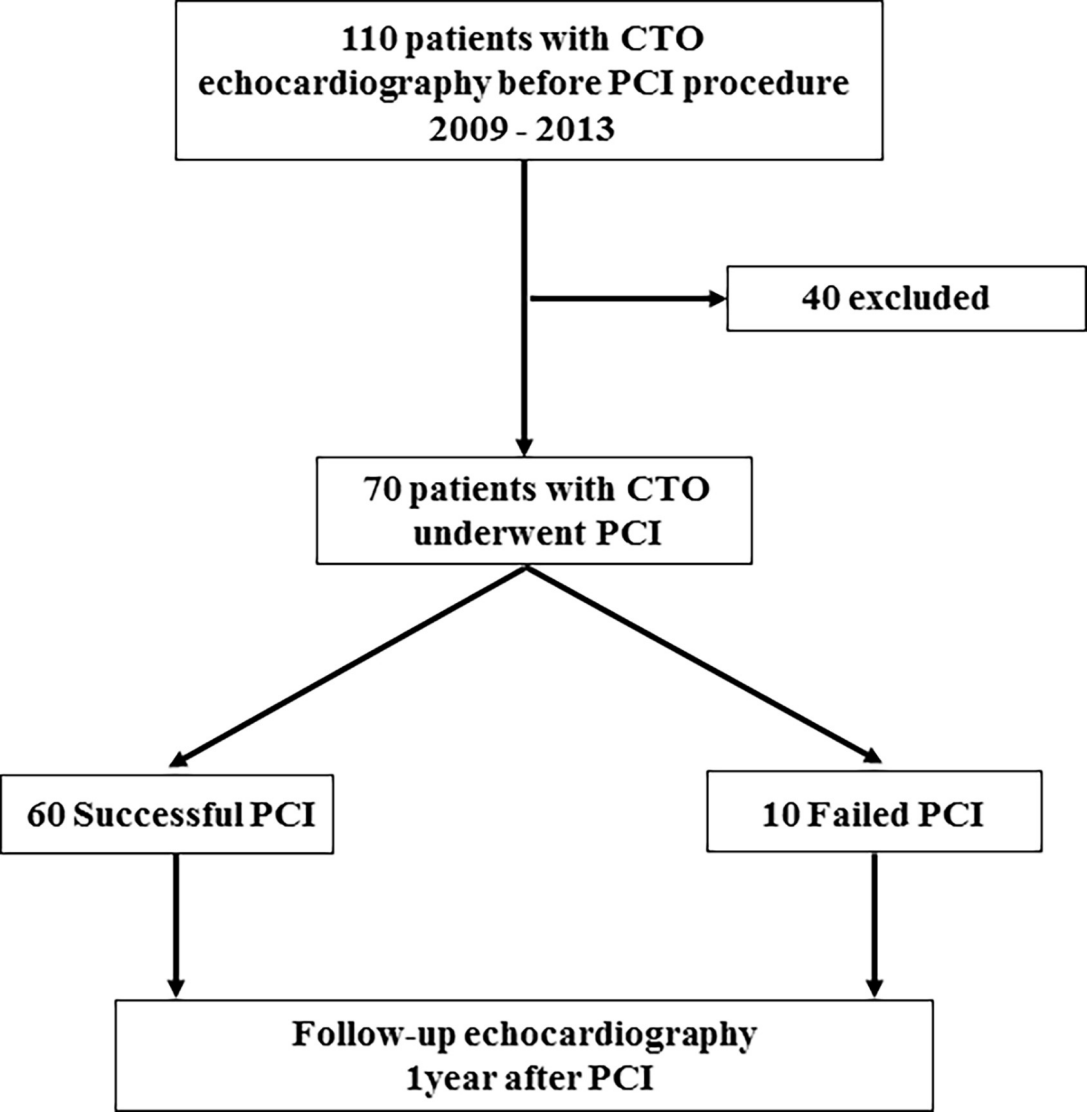
Patient flow chart.

Echocardiography was performed before and 9 months after the procedure (Fig 1). Comprehensive transthoracic echocardiography was performed by experienced research sonographers by using commercially available Aplio (Toshiba Medical Systems, Tokyo, Japan). Two-dimensional and color Doppler echocardiography were performed in standard parasternal and apical views. LV end-diastolic volume (EDV), end-systolic volume (ESV), and LVEF were measured using a modified Simpson method. All images were stored online and measured with offline software later by independent investigators who were blinded to the clinical data.

### LV strain measurements

**Fig 2:**
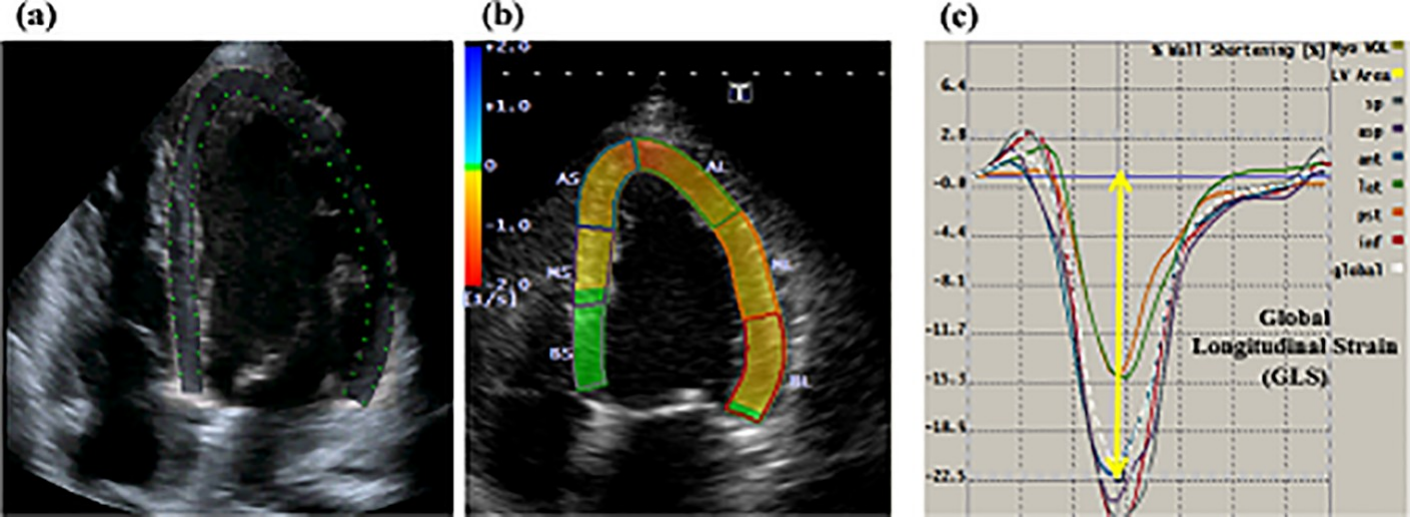
Example of the measurement of left ventricular (LV) global longitudinal strain (GLS) with a two-dimensional speckle-tracking echocardiography (2DSTE) in a patient with chronic total occlusion who underwent successful percutaneous coronary intervention (PCI). Figure (a) and (b) showed apical four-chamber view. White broken lines indicated the LV global strain curves for GLS. Yellow arrow indicated the mean LV global peak longitudinal strain; GLS: −22.5% (c).

The LV GLS was obtained by using 2DST software (Toshiba Medical Systems, Tokyo, Japan). The endocardial border was traced manually in the end-diastolic frame. The software automatically tracked the myocardium throughout the cardiac cycle. The peak values of segmental longitudinal strain were obtained from greyscale-recorded images in the apical four-chamber, two-chamber, and long-axis views with a frame rate between 50 and 70 frames per second, and GLS was obtained by averaging the peak values [17] (Fig 2). The coefficients of variation of interobserver variability for GLS was 8%. The coefficients of variation of intraobserver variability for GLS was 3% in our previous study [18].

### Statistical analysis

Data are expressed as mean ± standard deviation for continuous variables, and median and interquartile range for non-normally distributed continuous variables. Data from the successful and failed PCI groups were compared by using the Student’s t-test and one-way analysis of variance (ANOVA) for normally distributed continuous variables, the Wilcoxon–Mann–Whitney test for non-normally distributed continuous variables, and the χ2 test or Fisher’s exact test for categorical variables. Changes in the LVEDV, LVESV, LVEF and GLS data between the before and the 9 months after the procedure were assessed by ANOVA. A value of p <0.05 were considered statistically significant. MedCalc Version 12.7.8 (Acacialaan 22, B-8400 Ostend, Belgium) was used for all analyses.

## Results

Between January 2009 and December 2013, a total of 70 CTO lesions (in 70 patients) were targeted, and 60 lesions were successfully revascularized with PCI, and 10 lesions were failed (Fig 1). No procedural complications (coronary perforation, cardiac tamponade or emergent cardiac surgery) were observed in any patients undergoing CTO-PCI attempt. During the 9-month from the procedure, there were no changes in prescribed medical treatment, such as beta-blockers, angiotensin receptor blockers or angiotensin converting enzyme inhibitors, and nitrates, and no cardiac resynchronization therapy and pacemaker were implanted.

### Clinical and angiographic data

Baseline characteristics are shown in Table 1. The mean age of the patients was 65.1 ± 8.9 years. Eighty-four percent of the patients were male, 57% of the patients had a history of diabetes and 91% of the patients had hypertension. There were no significant difference in baseline characteristics between successful and failed PCI group. The angiographic characteristics of CTO lesions are shown in Table 2. The most common location of CTO was right coronary artery (RCA), followed by left anterior descending artery (LAD) and left circumflex artery (LCX). A total of 60 CTOs were revascularized, 19 CTOs in LAD, 31 CTOs in RCA, and 10 CTOs in LCX. Location of CTO and grade of collateral flow (Rentrop score) did not differ significantly between successful and failed PCI group. There were no stent thromboses during the 9 month follow-up.

**Table 1:** Patient baseline characteristics.

	All Patients (n = 70)	Successful PCI (n = 60)	Failed PCI (n = 10)	p Value
Demographics				
Age (years)	65.1 ± 8.9	65.2 ± 9.0	64.2 ± 8.6	0.74
Male, n (%)	59 (84)	51 (85)	8 (80)	0.90
Hypertension, n (%)	64 (91)	55 (92)	9 (90)	0.86
Diabetes Mellitus, n (%)	40 (57)	33 (55)	7 (70)	0.38
Dyslipidemia, n (%)	62 (89)	53 (88)	9 (90)	0.89
Smoking, n (%)	35 (50)	30 (50)	5 (50)	0.66
Systolic BP (mm Hg)	131 ± 24	132 ± 25	125 ± 18	0.45
Heart rate (beats/min)	68±11	67±11	71±14	0.30
Laboratory data				
Serum creatinine (mg/dL)	1.1 ± 1.0	1.1 ± 1.0	1.0 ± 0.7	0.66
Medications				
ACEI and/ or ARB, n (%)	41 (58)	35 (58)	6 (60)	0.92
Beta-blockers, n (%)	37 (53)	32 (53)	5 (50)	0.85
Nitrates, n (%)	27 (39)	22 (37)	5 (50)	0.65
Statin, n (%)	61 (87)	52 (87)	9 (90)	0.78

ARB, angiotensin II receptor blocker; ACEI, angiotensin converting enzyme inhibitor; BP, blood pressure; and PCI, percutaneous coronary intervention

**Table 2:** Lesion characteristics.

	All Patients (n = 70)	Successful PCI (n = 60)	Failed PCI (n = 10)	p Value
Chronic total occlusion location				
RCA/LCX/LAD, n (%)	37/11/22	31/10/19	6/1/3	0.79
Rentrop score (0/1/2/3)	0/1/60/9	0/1/51/8	0/0/9/1	0.89
Number of treated with stents, n (%)	N/A	57 (95)	N/A	N/A
Number of stents per lesion, n (%)	N/A	1.5 ± 0.5	N/A	N/A
Stent length, (mm. per stented lesion)	N/A	38 ± 16	N/A	N/A
Stent thrombosis, n (%)	N/A	0 (0)	N/A	N/A

LAD, left anterior descending artery; LCX, left circumflex artery; and RCA, right coronary artery; other abbreviations are as defined in Table 1.

### Echocardiographic measurement

**Fig 3:**
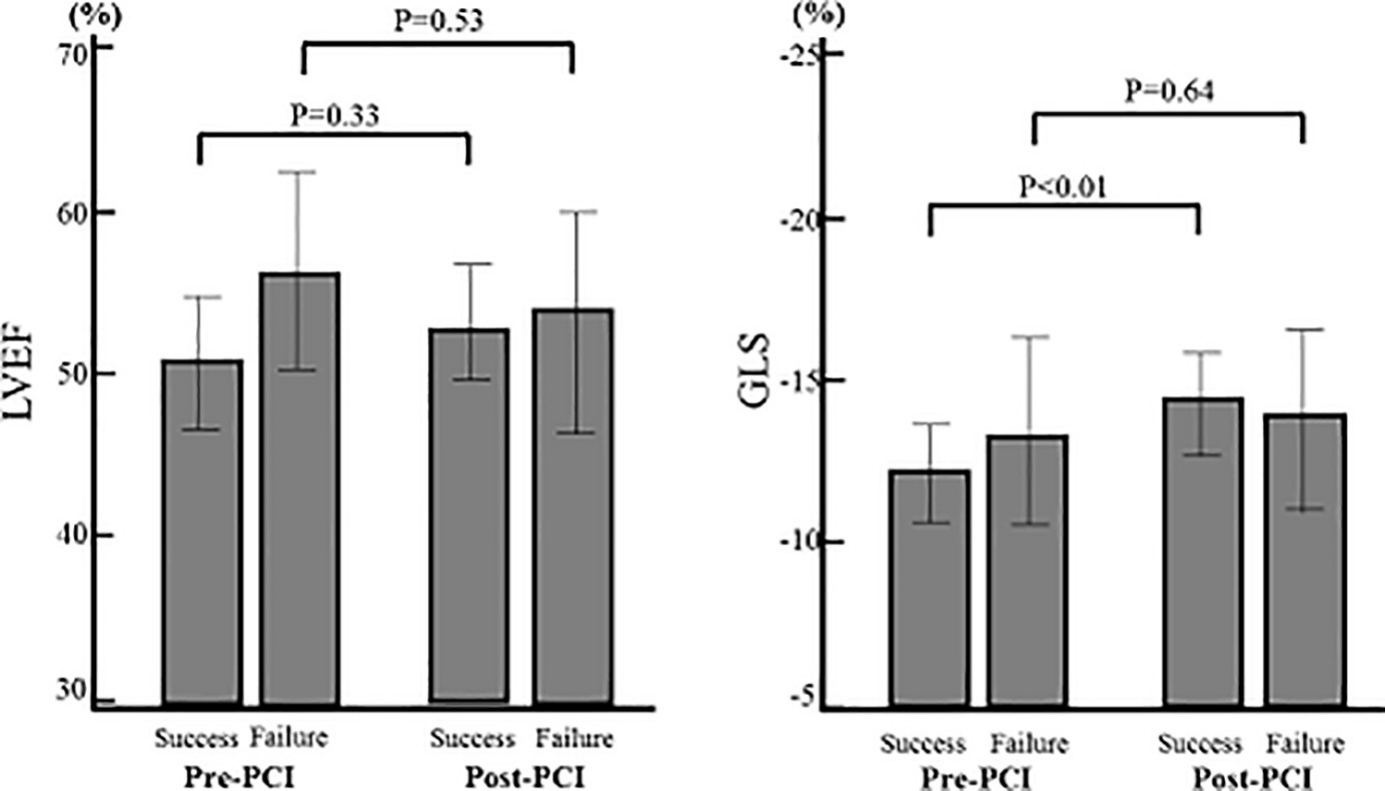
Change of LV function from baseline to 9 months after PCI. GLS in successful PCI group was significantly improved (P<0.01), whereas in failed PCI group that did not change significantly. LVEF showed no significant improvement in both successful and failed PCI groups.

**Fig 4:**
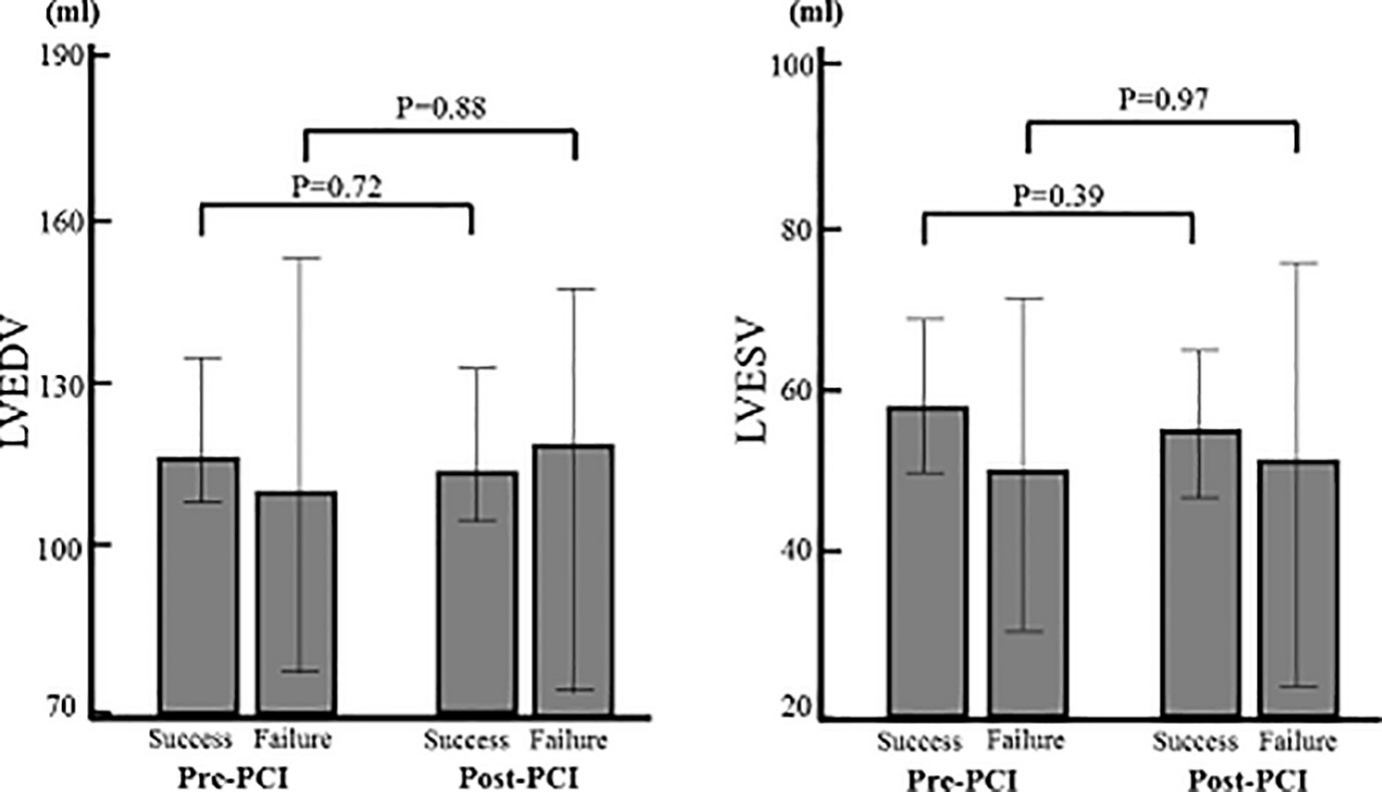
Change of LV volume from baseline to 9 months after PCI. LVEDV and LVESV showed no significant improvement in both successful and failed PCI groups.

Echocardiographic parameters are shown in Table 3. There were no significant differences in baseline LV function and volume between successful and failed PCI groups. GLS showed a significant improvement 9 months after successful PCI group (pre-PCI -12.4±4.1% vs. post-PCI -14.5±4.1%, P< 0.01), whereas that did not change significantly after failed PCI group (pre-PCI -13.2±4.2% vs. post-PCI -14.0±4.7%, P = 0.64). On the other hands, LVEF (pre-PCI 50.2±12.3% vs. post-PCI 52.4±10.7%, P = 0.33, pre-PCI 55.9±8.9% vs. post-PCI 53.2±9.9%, P = 0.53), LVEDV (pre-PCI 122.9±39.1ml vs. post-PCI 121.1±41.1ml, P = 0.72, pre-PCI 116.1±51.2ml vs. post-PCI 117.6±49.7ml, P = 0.88) and LVESV (pre-PCI 58.5±32.3ml vs. post-PCI 55.7±33.9ml, P = 0.39, pre-PCI 48.1±30.0ml vs. post-PCI 49.3±36.3ml, P = 0.97) showed no significant changes both successful and failed PCI groups during the 9 month follow-up, respectively (Figs 3 and 4).

**Table 3:** Echocardiographic measures.

	All Patients (n = 70)	Successful PCI (n = 60)	Failed PCI (n = 10)	p Value
Echocardiographic data				
LVEDV (mL)	121.9 ± 40.7	122.9 ± 39.1	116.1 ± 51.2	0.63
LVESV (mL)	57.0 ± 31.9	58.5 ± 32.3	48.1 ± 30.0	0.35
LVEF (%)	51.0 ± 12.0	50.2 ± 12.3	55.9 ± 8.9	0.11
E/A ratio	0.72 ± 0.20	0.72 ± 0.19	0.73 ± 0.21	0.85
GLS (%)	-12.2 ± -4.7	-12.4 ± -4.1	-13.2 ± -4.2	0.47

E/A ratio, ratio of early transmitral flow to atrial contraction; GLS, global longitudinal strain; LVEF, left ventricular ejection fraction; LVEDV, left ventricular end-diastolic volume; and LVESV, left ventricular end-systolic volume; other abbreviations are as defined in Tables 1 or 2.

## Discussion

In this study, we investigated the impact of revascularization of CTO on LV function using LV GLS. Successful PCI for CTO improved LV function assessed by LV GLS. To the best of our knowledge, this is the first study to demonstrate the comparison with successful and failed CTO-PCI patients with a positive functional ischemia before PCI, according to LV function evaluated with LV GLS before and after 9 month PCI.

CTO is common, being reported in 30% of patients undergoing coronary angiography [19, 20]. However, the benefits of PCI for CTO are controversial. The procedure lasted several hours with significant radiation exposure and contrast dose. Furthermore the higher complication rates of CTO procedure even in experienced dedicated PCI operators and the higher failure rates of CTO-PCI, were still reported [21–26]. Therefore, it is important to select the patients suitable for CTO-PCI and to assess the impact of CTO revascularization by conventional assessment.

Several investigators reported the effect of revascularization of CTO on LV function. Henriques et al. reported that they did not find an overall benefit for CTO-PCI in terms of LVEF or LVEDV [27]. Mashayekhi et al. reported that no benefit was seen for CTO-PCI in terms of the segmental wall thickening, LVEF and LVEDV [28]. In this study, LVEF and LVEDV also showed no significant changes both successful and failed PCI groups during the 9 month follow-up, respectively. However, since there was report that reduced major adverse coronary event rates at 12 months in CTO-PCI compared with optimal medical therapy [28], we assessed cardiac function evaluated by GLS, with the idea that some improvement in cardiac function that can not be assessed by LVEF or LVEDV in successful CTO-PCI group.

GLS assessed with 2DSTE is a new echocardiographic technique for the evaluation of global myocardial function. This method is based on tracking the characteristics speckle patters created by interference of ultrasound beams in the myocardium. Its accuracy has been confirmed using CMR [29] and it does not require special equipment. Kalam et al. showed that the subendocardial longitudinal fiber is more sensitive to myocardial ischemia and the depressed LV longitudinal function occured at an earlier stage than abnormal radial contraction, as assessed by the LVEF [30]. GLS represents the subendocardial longitudinal fiber movement [31], therefore, in the case of preserved LVFE CTO patients, GLS assessed by 2DSTE is a quite suitable method to evaluate the change of myocardial ischemic burden.

In this study, we studied of preserved LVEF patients with positive functional ischemia of CTO lesion before PCI, and assessed the effect of revascularization of CTO on LV function using GLS assessed by 2DSTE before and 9months after procedure. GLS showed a significant improvement 9 months after successful PCI, whereas that in failed PCI group did not change significantly. These results indicated that CTO revascularization improves LV function.

### Limitation

This study is limited by the fact that it was a single center, retrospective study and had a small sample size, which might diminish the power of the drawn statistical inference. A further study with a large scale prospective, multicenter design is thus required to confirm our results. An additional limitation to this study is that radial and circumferential strain were not explored. However, it has been recently reported that longitudinal deformation may be a more sensitive marker of cardiac function compared with radial and circumferential strain, especially in chronic ischemic patients [32], so not assessing the radial and circumferential strain dose not affect the result. And then the assessment of the success or failure of the PCI was done immediately after the PCI; it may not reflect what happened later during the 9 months.

## Conclusion

Successful PCI for CTO improved LV function, assessed by LV GLS.
